# Dynamic-Parameterized Reconstruction Model for Resource-Aware Spatial Intelligence

**DOI:** 10.3390/s26082355

**Published:** 2026-04-11

**Authors:** Hongyi Huang, Yanni Zhang, Liang Song, Zhen Zhao, Xiaopeng Yang

**Affiliations:** 1 College of Intelligent Robotics and Advanced Manufacturing, Fudan University, Shanghai 200433, China; 23210860046@m.fudan.edu.cn; 2 Shanghai East-Bund Institute on Networking Systems of AI, Shanghai 202162, China; zyn@fudan.edu.cn; 3 China State Construction Engineering (Hong Kong) Limited, Hong Kong, China; zhen_zhao@cohl.com (Z.Z.); yangxiaopeng@cohl.com (X.Y.)

**Keywords:** monocular 3D reconstruction, early exit, resource-aware inference, spatial intelligence

## Abstract

Spatial intelligence in autonomous driving requires object-level 3D geometry, yet existing monocular mesh reconstruction methods usually operate with a fixed inference path and a single mesh parameterization, which limits their flexibility under heterogeneous resource constraints. To address this issue, we propose DyPRSI, a dynamic-parameterized framework for monocular vehicle 3D reconstruction that provides multiple predefined accuracy–latency operating points within a single model. DyPRSI inserts two early exits into a shared Res2Net–BiFPN trunk and associates each exit with an exit-specific mesh specification, forming a coarse-to-fine reconstruction hierarchy across network depth. To better match the efficiency requirements of shallow branches, DyPRSI adopts lightweight coordinate-classification keypoint decoding for EE1 and EE2, while retaining a heatmap-regression keypoint head in the Main branch to preserve the upper bound of reconstruction accuracy. Experiments on ApolloCar3D show that DyPRSI-Main achieves competitive reconstruction performance, whereas EE1 and EE2 substantially reduce end-to-end inference latency and provide useful alternatives under different resource requirements. Ablation studies further show that the speedup mainly comes from the lightweight branch-specific keypoint heads, while the exit-specific mesh settings help organize stable coarse-to-fine reconstruction behavior across branches. These results indicate that DyPRSI is a practical monocular vehicle reconstruction framework for resource-aware spatial intelligence.

## 1. Introduction

With the rapid progress of autonomous driving and mobile robotics, the perceptual demands of embodied agents are shifting from conventional 2D image understanding toward spatial intelligence. Spatial intelligence emphasizes an agent’s ability to continuously build, maintain, and exploit a world model that integrates both geometric and semantic information, thereby supporting localization, navigation, scene understanding, and decision-making [1]. In practice, such systems must deliver accurate and stable environmental representations while simultaneously satisfying stringent real-time constraints under limited on-board compute. This makes it essential to balance representational capacity against computational cost when designing perception algorithms.

Within a spatial-intelligence stack, 3D scene representations constitute a fundamental building block for world modeling [2,3]. Compared with 2D image cues, 3D representations explicitly encode geometric structures and provide more robust support for tasks such as scene understanding, interaction reasoning, and simulation. Among common 3D representations, triangular meshes are particularly attractive for autonomous-driving applications because they preserve topology, offer compact structured geometry, and interface naturally with rendering and physics engines.

Despite their advantages, most existing monocular mesh reconstruction methods follow a fixed inference pipeline and produce outputs with a single geometric specification. Once trained, these models typically operate at a fixed network depth and output a fixed mesh specification, which limits their flexibility for different deployment requirements. In practice, however, autonomous-driving systems often face heterogeneous resource constraints. Some deployment settings may prioritize higher geometric fidelity for detailed object understanding, whereas others may favor lower-latency predictions with reduced reconstruction complexity. This mismatch motivates a reconstruction framework that can support multiple structured operating points within a single model, instead of relying on a fixed computational path and a fixed mesh specification.

A direct extension of early-exit inference to monocular 3D reconstruction is not straightforward. Unlike classification or simple detection tasks, vehicle mesh reconstruction requires structured outputs that jointly involve object pose, keypoints, and mesh geometry. Therefore, exiting at different depths is not merely a matter of truncating backbone computation, it also requires matching each branch with an appropriate geometric output scale and branch-specific prediction design. This observation motivates us to move beyond a naive early-exit formulation and instead build a multi-exit structured reconstruction framework with exit-specific output specifications.

To this end, we propose DyPRSI, a dynamic-parameterized monocular vehicle 3D reconstruction framework for resource-aware spatial intelligence. DyPRSI inserts two early exits (EE1 and EE2) into a shared Res2Net-BiFPN trunk and associates each exit with a predefined mesh specification, forming a coarse-to-fine hierarchy across branches. In addition, to better match the efficiency requirements of shallow branches, we adopt lightweight coordinate-classification keypoint decoding in EE1 and EE2, while retaining heatmap-regression keypoint estimation in the Main branch to preserve the accuracy upper bound. In this way, a single model can provide multiple predefined accuracy–latency operating points for deployment under different resource requirements, without the need for adaptive online exit selection during inference. The main contributions are as follows:

- We propose a multi-exit monocular vehicle 3D reconstruction framework that maintains valid pose-and-mesh outputs at all exits and provides multiple predefined accuracy–latency operating points within a single model, making it suitable for resource-aware spatial intelligence.
- We introduce an exit-specific dynamic parameterization strategy by binding each exit to a predefined mesh specification, thereby coupling branch depth with geometric representation scale and enabling coarse-to-fine structured reconstruction across branches.
- We design branch-dependent keypoint heads for different exits, with lightweight coordinate-classification decoding adopted for low-latency early exits and heatmap regression retained in the Main branch to preserve the accuracy ceiling, effectively reducing RoI-head latency without sacrificing the strong performance of the full branch.

## 2. Related Work

### 2.1. Spatial Intelligence and Spatial Representations

Spatial intelligence is commonly used to describe an embodied agent’s ability to perceive, model, reason about, and interact with the physical world. The central requirement is to construct spatial representations that support downstream reasoning and decision-making. Existing studies have explored a broad spectrum of representations spanning geometric and semantic forms, including point clouds [4], voxels [5], implicit fields [6], and object-centric structured representations [7].

In traffic scenarios, spatial representations are often expected to simultaneously satisfy geometric fidelity, structural interpretability, and compatibility with downstream modules. For this reason, object-level and structured representations of 3D shape and pose are particularly attractive [8,9]. However, stronger representational capacity typically comes with higher computational cost and longer inference pipelines, which makes it difficult to meet strict latency constraints on resource-limited platforms. This practical tension has motivated research on spatial representation learning methods that can produce elastic outputs under deployment constraints.

Beyond perception itself, recent autonomous-driving studies have also highlighted that representation quality directly affects downstream control and decision-making performance. Recent efforts on autonomous eco-driving, reinforcement learning-based highway vehicle control, and end-to-end decision-making in challenging curved-road scenarios [10,11,12] further indicate that spatial representations in driving systems should not only preserve geometric expressiveness, but also remain compatible with resource-constrained deployment and downstream reasoning. This perspective further motivates resource-aware object-level 3D representation learning for spatial intelligence.

### 2.2. Monocular 3D Reconstruction for Traffic Scenarios

Monocular 3D reconstruction is appealing for autonomous driving due to its low cost and flexible deployment. Yet, because monocular cues suffer from depth and scale ambiguities, performance depends heavily on the design of priors and constraints. Existing object-level monocular reconstruction methods can be roughly organized along three lines. Some rely on 2D geometric constraints, such as keypoints or silhouettes [13], others reconstruct meshes via templates or deformable models [14], and a third group uses implicit representations or generative modeling [15].

Compared with implicit fields or high-resolution volumetric rendering, parametric meshes provide a more compact way to output structured shapes, and interface naturally with metric-scale reasoning, pose estimation, and other downstream components. For example, Deep MANTA [16] adopts a coarse-to-fine multi-task framework to estimate mesh vertices and object pose, GSNet [17] follows a divide-and-conquer strategy and generates meshes by fusing multiple PCA bases, and BAAM [18] factorizes the shape prior into a mean shape and template offsets, enabling more detailed mesh reconstruction. Despite steady progress in accuracy, these methods typically produce meshes with fixed parameterization, which limits their ability to accommodate the diverse compute and latency budgets encountered in real-time systems.

### 2.3. Elastic Inference Under Resource Constraints

To satisfy real-time requirements in practical deployments, dynamic inference and conditional computation aim to trade accuracy for latency by allocating computation on demand. Representative directions include anytime prediction [19,20,21] and early-exit networks [22,23], which have developed into relatively mature research lines. Early-exit methods typically attach auxiliary prediction heads to shallow layers and, during inference, terminate computation early based on confidence estimates or budget constraints, thereby reducing computation and latency.

This idea was initially explored in classification settings. For example, Panda et al. [24] explored early-exit inference to reduce power consumption in pattern recognition, and BranchyNet [25] implemented this idea by inserting multiple early-exit branches into networks such as LeNet [26] and AlexNet [27]. In recent years, early-exit strategies have been widely applied to tasks including image classification [28], speech recognition [29,30], and graph neural networks [31]. However, most existing studies focus on relatively simple outputs such as class labels or detections, while applications to high-dimensional, structured outputs such as 3D reconstruction remain comparatively underexplored.

Beyond early-exit prediction, resource-aware inference has also been studied through elastic network design and input-adaptive computation. Slimmable Neural Networks enable a single model to operate at multiple widths and provide runtime accuracy–efficiency trade-offs, while Dynamic Slimmable Network further introduces input-dependent width adjustment for hardware-efficient dynamic inference [32,33]. At a finer granularity, Dynamic Convolution performs input-adaptive aggregation of multiple convolution kernels to improve representational capacity under constrained computation [34]. More recently, Stitchable Neural Networks extend this line by constructing flexible deployment configurations from anchor models with different complexity–performance trade-offs [35]. However, most of these methods are primarily developed for image classification or backbone-level adaptation. In contrast, structured monocular 3D reconstruction requires jointly predicting pose, keypoints, and mesh geometry, so dynamic inference in this setting must preserve output validity and geometric consistency across branches. This difference motivates our exit-specific mesh specification and branch-dependent prediction heads, rather than directly adopting standard elastic network designs.

## 3. Methods

### 3.1. Model Overview

**Figure 1:**
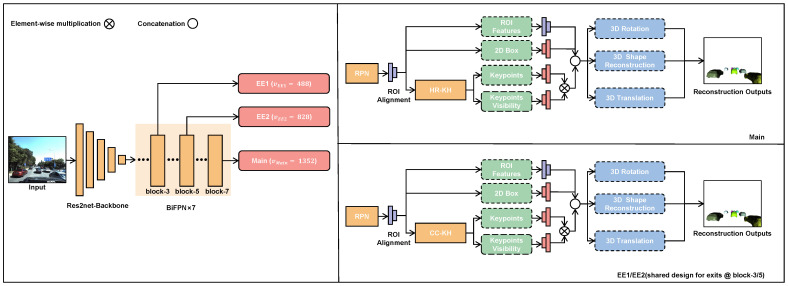
Overview of DyPRSI. A shared Res2Net–BiFPN trunk attaches two early-exit branches at BiFPN blocks 3 and 5, and a Main branch at block 7. The three branches output meshes with 488, 828, and 1352 vertices to enable elastic accuracy–latency trade-off. At each exit branch, Region Proposal Network (RPN) and RoIAlign extract RoI features, which are fused with keypoint confidence and fed to the rotation, translation, and shape reconstruction heads. The Main branch uses a heatmap-regression keypoint head, while EE1/EE2 use a lightweight coordinate-classification head based on SimCC.

An overview of DyPRSI is illustrated in Figure 1. We build an integrated detection-reconstruction network on top of the Mask R-CNN [36] framework. Specifically, a Res2Net [37] backbone is first adopted to extract multi-scale features from the input image. These features are then fused across scales by stacking seven BiFPN [38] blocks, producing multi-level representations that jointly encode semantic context and spatial localization. To enable an elastic accuracy–latency trade-off, we introduce two early-exit branches at the outputs of the 3rd and 5th BiFPN blocks, denoted as EE1 and EE2, while using the output of the 7th BiFPN block as the default high-accuracy Main branch. Each exit b∈{EE1,EE2,Main} is bound to a predefined mesh specification $v_{b}$, which is defined here as the vertex budget of the output mesh parameters $R^{3v_{b}}$ (instantiated as $v_{EE1}=488$, $v_{EE2}=828$, and $v_{Main}=1352$). All exits share the same backbone and BiFPN trunk, but detach exit-specific prediction heads to accommodate different computational budgets.

Within each exit, candidate bounding boxes are generated by the detection and 2D parsing heads. We then apply Region of Interest Align (RoIAlign) to extract the corresponding RoI features and explicitly predict keypoints along with their visibility. Consequently, the 3D reconstruction stage relies on three types of 2D cues: 2D bounding boxes, RoI features, and keypoints with visibility. The three exits differ only in the complexity of the 2D parsing heads and the mesh specification $v_{b}$ of the mesh output. Main employs a standard heatmap-regression keypoint head (HR-KH) and uses a larger parameter scale $v_{Main}$, yielding higher geometric fidelity. In contrast, EE2 and EE1 adopt lightweight coordinate-classification keypoint heads (CC-KH) and use smaller scales $v_{EE2}$ and $v_{EE1}$, respectively, to reduce RoI-head latency. During inference, different exits can be used as predefined operating points according to deployment requirements, thereby providing flexible accuracy–latency trade-offs within a unified framework.

### 3.2. Dynamic-Parameterized Multi-Resolution Mesh Reconstruction

To accommodate varying compute budgets and latency constraints, we formulate vehicle mesh reconstruction as a topology-preserving, parameterized deformation problem. Specifically, given a shape-prior template, the network parses 2D cues from the input features and predicts instance-specific deformation parameters, which are then applied to the template to obtain the final mesh. Furthermore, we equip different exits with shape-prior template sets matched to their mesh specification $v_{b}$. This design exposes a coarse-to-fine spectrum of mesh parameter dimensionalities within a unified, topology-preserving deformation framework, enabling an adjustable trade-off between geometric detail and computational cost.

For each exit *b*, we build a set of shape-prior templates from the 79 vehicle CAD meshes provided by ApolloCar3D [39]. Concretely, we leverage the differentiable rendering and optimization pipeline of SoftRas [40] to deform a sphere with fixed topology and mesh specification $v_{b}$ (i.e., the vertex budget) to fit each category CAD mesh, resulting in three template sets instantiated at $v_{EE1}=488$, $v_{EE2}=828$, and $v_{Main}=1352$. We assign these scale-matched priors to EE1, EE2, and Main respectively, encouraging exits at different depths to model geometric details at the corresponding parameter scales.

### 3.3. Attention-Guided Modeling Mechanism

Following BAAM’s attention-guided modeling (AGM) [18], we factorize object shape into a mean template ${\bar{M}}_{s}\in R^{3v}$, a template-level offset $O_{s}\in R^{p\times 3v}$, and an instance-specific offset predicted from RoI features. The final shape of the target instance can be expressed as

(1) $$M={\bar{M}}_{s}+AO_{s}+O_{o}.$$

where $A\in R^{n\times p}$ denotes the attention weight matrix, whose each row represents the contribution of each template to the corresponding object shape. Accordingly, the key objective of shape-aware attention is to jointly estimate the attention scores *A* and the instance offset $O_{o}$ based on the correlation between the object and the shape priors, thereby enabling instance-level shape recovery under prior constraints.

**Figure 2:**
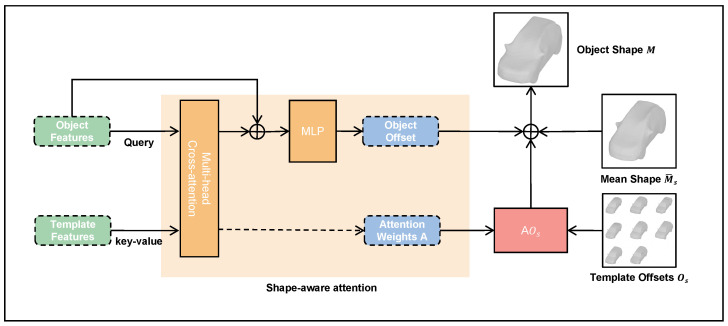
Attention-guided modeling mechanism. The reconstruction head combines object features and embedded template features through multi-head cross-attention to produce attention weights and instance-specific offsets, which are then used for prior-driven mesh deformation.

The AGM-based reconstruction head used in DyPRSI is illustrated in Figure 2. Building upon the above AGM formulation, we instantiate AGM as the reconstruction head for all three exits (EE1, EE2, and Main), enabling selectable accuracy–latency paths. For each exit *b*, we configure a mean shape and template offsets that match its mesh specification $v_{b}$, i.e., ${\bar{M}}_{s}^{\left(b\right)}\in R^{3v_{b}}$ and $O_{s}^{\left(b\right)}\in R^{p\times 3v_{b}}$. Accordingly, each exit performs prior-driven shape recovery under the same AGM structure, while operating at different parameter scales, and derives an object representation from the exit features. During inference, each exit runs the AGM module on its own, producing its attention weights and instance offsets.

(2) $${\tilde{O}}_{o}=X_{o}+\mathrm{MCA}\left(\mathrm{LN}\left(X_{o}\right),X_{s}\right).$$

(3) $$O_{o}=\mathrm{MLP}\left({\tilde{O}}_{o}\right).$$

Here, *v* denotes the vertex budget of the current branch, *p* is the number of shape templates, and *n* is the number of object instances in a batch. The object feature $X_{o}$ is composed of the 2D bounding box, RoI features, and keypoint information augmented with visibility cues. Due to differences in the input feature hierarchy and the complexity of the exit-specific 2D parsing heads, the three exits yield distinct object representations. The template features $X_{s}$ are obtained by passing the exit-specific template offsets through a learnable embedding, which improves the discriminability of template representations and encourages reconstruction heads at different parameter scales to focus on geometric detail patterns at the corresponding scales. Following BAAM [18], we use MCA(·,·) to measure object–template correlation and produce attention scores, where LN(·) and MLP(·) denote layer normalization and a multilayer perceptron respectively. In Equation (2), ${\tilde{O}}_{o}$ is the intermediate fused representation, and Equation (3) maps it to the final instance-specific offset $O_{o}$ for prior-driven shape recovery. At inference time, the reconstruction result can be taken from a selected exit configuration, allowing the framework to provide multiple predefined accuracy–latency operating points.

### 3.4. Keypoint Localization and Visibility Prediction

To introduce stable structural constraints under the monocular setting, we explicitly predict vehicle keypoints and their visibility from RoI features, and use them as auxiliary cues for subsequent 3D pose and shape regression. Specifically, given the proposal boxes produced by the detection branch and the corresponding region features extracted via RoIAlign, the keypoint module outputs the 2D locations and visibility confidences of J=66 keypoints:

(4) $$\hat{K}={\left\{\left({\hat{x}}_{j},{\hat{y}}_{j},{\hat{v}}_{j}\right)\right\}}_{j=1}^{J}.$$

where ${\hat{v}}_{j}$ indicates the visibility of the keypoint under the current viewpoint.

To enable an elastic accuracy–latency trade-off, we employ keypoint heads of different complexities across exit-specific heads: EE1 and EE2 adopt SimCC [41] to reduce inference cost, while Main follows the heatmap-based keypoint head used in the BAAM baseline [18] to preserve the upper bound of accuracy. Both heads are unified at the output as $\left({\hat{x}}_{j},{\hat{y}}_{j},{\hat{v}}_{j}\right)$, facilitating reuse by downstream modules.

Coordinate-Classification Keypoint Head. The early exits (EE1/EE2) target low-latency scenarios. Instead of predicting dense 2D heatmaps, SimCC decomposes each keypoint into two lightweight 1D coordinate-classification problems along the x and y axes. This avoids the extra computation of high-resolution heatmap generation and subsequent resizing, making it more suitable for shallow exits with strict latency requirements. Therefore, we adopt SimCC to reformulate 2D keypoint localization as the prediction of two 1D distributions along the x and y axes, avoiding the extra computation incurred by generating high-resolution 2D heatmaps and their upsampling. Concretely, we map each keypoint’s relative position within a proposal box onto a fixed output grid, and further refine the discretized coordinates into 1D bins of length 112. During training, we use 1D Gaussian soft labels to mitigate quantization error, and align the predicted distributions with the soft labels using a log-softmax-based Kullback–Leibler (KL) divergence. In addition, we leverage the visibility annotations provided by the dataset to mask or down-weight invisible keypoints, preventing them from dominating the learning process.

At inference time, we take the peak locations in the 1D x/y distributions, recover the coordinates within the proposal box, and map them back to the image plane to obtain $\left({\hat{x}}_{j},{\hat{y}}_{j}\right)$. The keypoint confidence ${\hat{v}}_{j}$ is defined as the minimum of the peak probabilities along the two axes, so that the decoded output remains compatible with the unified keypoint interface $\left({\hat{x}}_{j},{\hat{y}}_{j},{\hat{v}}_{j}\right)$ used by downstream pose and shape regression.

Heatmap-Regression Keypoint Head. For the Main branch, we follow the Keypoint R-CNN [36] head adopted in the BAAM baseline [18] to preserve the upper bound of accuracy. This head predicts a 2D heatmap for each keypoint. During training, keypoints are quantized onto discrete locations on the RoI grid, and a classification-style cross-entropy loss is computed for visible keypoints.

During inference, the heatmaps are resized to match the proposal box scale via interpolation, the peak location is taken as the keypoint coordinate, and the normalized peak response is used as the keypoint confidence.

### 3.5. Loss Functions

For each exit b∈{EE1,EE2,Main} (with $v_{b}$ vertices), all losses are computed based on the predictions and ground truth of *b*.

Detection loss. The detection loss penalizes inaccurate 2D predictions:

(5) $$L_{\det}=L_{\mathrm{rpn}}+L_{\mathrm{bbox}}+L_{\mathrm{kpts}}.$$

where $L_{\mathrm{rpn}},L_{\mathrm{bbox}},L_{\mathrm{kpts}}$ follow the standard RPN, box-regression, and keypoint losses as in Mask R-CNN [36].

Regression loss. The translation loss is defined as

(6) $$L_{\mathrm{trans}}=|x-\hat{x}|+|y-\hat{y}|+\frac{\sqrt{2}}{u}\left|z-\hat{z}\right|+\log\left(u\right).$$

where $\hat{x},\hat{y},\hat{z}$ denote the ground-truth translation and *u* denotes the depth-uncertainty scalar predicted by the network. The last two terms constitute an uncertainty regression loss [42,43], which allows the model to assign higher uncertainty *u* to hard cases or samples with noisy depth cues, thereby alleviating training instability.

Rotation is supervised with a periodic wrapped-L1 objective $L_{\mathrm{rot}\;}$ to properly handle angle wrap-around, keeping the residual within [−π,π]:

(7) $$L_{\mathrm{rot}}=\left\{\begin{array}{cc} \left|p_{r}-{\hat{p}}_{r}\right|, & \left|p_{r}-{\hat{p}}_{r}\right|\leq \pi \\ \left|2\pi -\left|p_{r}-{\hat{p}}_{r}\right|\right|, & \left|p_{r}-{\hat{p}}_{r}\right|>\pi . \end{array}\right.$$

The shape loss uses an L2 distance between the predicted mesh vertices $M\in R^{3v_{b}}$ and the ground-truth mesh $\hat{M}\in R^{3v_{b}}$:

(8) $$L_{\mathrm{shape}}={∥M-\hat{M}∥}_{2}^{2}.$$

3D space loss. Since translation, rotation, and shape are coupled in 3D space, we reuse BAAM’s 3D space loss [18] and apply it to the three transformed vertex sets to enforce structural consistency among them. Let the predicted mesh at exit *b* be $M\in R^{3v_{b}}$ and reshape it into $M_{*}\in R^{3\times v_{b}}$. With $R\in R^{3\times 3}$ and translation *t*, we transform the predicted vertices into three frames (full world, rotation-only, and translation-only) and compute the corresponding 3D space penalties in each frame:

(9) $$M_{\mathrm{world}}=RM_{*}+t.$$

(10) $$M_{\mathrm{rot}}=RM_{*}.$$

(11) $$M_{\mathrm{trans}}=M_{*}+t.$$

The 3D space loss is then defined as

(12) $$L_{3D}=\sum_{s\in \{\mathrm{world},\mathrm{rot},\mathrm{trans}\}}{∥M_{s}-{\hat{M}}_{s}∥}_{1}.$$

In Equation (6), *x*, *y*, and *z* denote the predicted translation components, whereas $\hat{x}$, $\hat{y}$, and $\hat{z}$ are the corresponding ground-truth values. The scalar *u* is the predicted depth uncertainty. In Equation (7), $p_{r}$ and ${\hat{p}}_{r}$ denote the predicted and ground-truth yaw angles, respectively. In Equations (9)–(12), *R* and *t* denote the predicted rotation matrix and translation vector, and ${\hat{M}}_{s}$ denotes the ground-truth vertices transformed into the same frame s∈{world,rot,trans} as $M_{s}$.

Total Loss. For each exit, the total loss is defined as

(13) $$L_{b}=L_{\det}+L_{\mathrm{trans}}+L_{\mathrm{rot}}+L_{\mathrm{shape}}+L_{3D}.$$

To enable joint training of multiple exits, we take a weighted sum of the losses from all three exits:

(14) $$L_{\mathrm{total}}=\sum_{b\in \{\mathrm{EE}1,\mathrm{EE}2,\mathrm{Main}\}}\lambda_{b}L_{b}.$$

where $\lambda_{b}$ balances the gradient contributions of different exits during training.

## 4. Experiments

### 4.1. ApolloCar3D Dataset

Dataset Overview. We conduct training and evaluation on the ApolloCar3D [39] dataset. Built upon the ApolloScape autonomous-driving corpus, ApolloCar3D was curated by selecting 5277 driving-scene images from approximately 200,000 publicly released images in the semantic segmentation track. The selection favors scenes with high vehicle density, diverse appearances, and broad coverage of driving environments, including highways, urban roads, and intersections. Each image contains 11.7 vehicle instances on average (up to 37), and each instance provides 2D keypoints together with 3D pose parameters, which consist of translation and rotation labels, providing rich supervision for vision-based autonomous-driving algorithms and systems. In addition, ApolloCar3D provides industrial-grade 3D computer-aided design (CAD) models for 79 common vehicle types, together with their real-world dimensions and semantically defined keypoints; every vehicle instance in the images is matched to one CAD model from this set.

Evaluation Metrics. ApolloCar3D follows an instance average precision protocol analogous to object detection, while redefining a “correct match” by extending the conventional 2D/3D bounding-box intersection-over-union (IoU) criterion to a joint constraint over shape, translation, and rotation, which better reflects the objective of 3D vehicle instance understanding. Shape similarity is measured via multi-view projected silhouette consistency: 100 viewpoints are sampled along the yaw direction, the predicted shape is projected onto a 1280 × 1280 image plane, and the IoU between projected silhouettes is computed for each view; the mean IoU over views serves as the shape score. Translation and rotation errors are evaluated using metrics commonly adopted in autonomous driving and camera pose estimation, and together with the shape requirement form the true-positive criterion.

To avoid the coarseness of a single threshold, ApolloCar3D adopts an MS COCO-style evaluation by computing instance-level 3D average precision, A3DP, over 10 threshold sets ranging from lenient to strict, jointly assessing translation, rotation, and shape accuracy. For clarity and fair comparison, we mainly report two representative settings in our experiments: the lenient criterion (c-l) and the strict criterion (c-s). During evaluation, only vehicle instances with depth less than 100 m are considered. Moreover, motivated by the higher sensitivity to near-range errors in driving scenarios, the original benchmark also introduces a relative translation error variant (analogous to AbsRel), leading to A3DP-Abs and A3DP-Rel, which enforce absolute and relative translation constraints, respectively.

ApolloCar3D is selected in this work because it provides a task-aligned benchmark for object-level monocular vehicle mesh reconstruction, including CAD-aligned vehicle templates, keypoint supervision, pose annotations, and A3DP-based evaluation. These annotations are directly compatible with our topology-preserving, prior-driven mesh reconstruction setting. We acknowledge that validation on additional benchmarks would be valuable for assessing broader generalization, and leave cross-dataset evaluation as future work.

### 4.2. Implementation Details

We adopt a Res2Net [37] backbone pre-trained on the COCO [44] dataset and perform two-stage training on a single RTX 4090 GPU. In the first stage, we train for 10 epochs using only the detection loss (AdamW, learning rate $1\times 10^{-4}$) to stabilize the proposal generation and RoI features. In the second stage, we enable the full objective and continue training for 30 epochs. Unlike the single-exit setting, the second stage jointly trains the three exits (EE1/EE2/Main): the heads attached to BiFPN block-3/5/7 are all executed in each iteration to compute their respective losses, which are then aggregated via a weighted sum. Gradients are jointly back-propagated to update the shared backbone and BiFPN, while each exit-specific head is updated only by its corresponding loss. Training adopts batch size 1, and the learning rate begins at $1\times 10^{-4}$ and drops tenfold at epoch 20. For implementation consistency and fair comparison, we re-train GSNet and BAAM on ApolloCar3D under the same optimizer and learning-rate policy. Detailed experimental environment parameters are shown in Table 1.

### 4.3. Main Results

Table 2 reports the main results of DyPRSI on ApolloCar3D. Built on a shared trunk (backbone + BiFPN), our model introduces two early-exit branches (EE1/EE2) at different BiFPN depths together with a high-accuracy Main branch (Main), forming an elastic accuracy–latency trade-off. Note that Inference Time is measured as the end-to-end forward latency, covering backbone + BiFPN + RoI heads. The number of mesh vertices only affects post-processing cost and therefore does not change the reported forward inference time.

In terms of accuracy, DyPRSI-Main achieves 23.39 on A3DP-Abs, essentially matching BAAM (23.40), which indicates that our Main branch preserves a competitive high-accuracy ceiling. On A3DP-Rel, DyPRSI-Main reaches 21.83, a clear improvement over BAAM (19.39). For the early-exit branches, DyPRSI-EE2 attains 22.57/19.63 on A3DP-Abs/A3DP-Rel, remaining close to the Main branch while substantially reducing latency. DyPRSI-EE1 further cuts computation and still achieves 21.31 on A3DP-Abs, suggesting that even the most lightweight path maintains a usable reconstruction quality. Overall, the three branches exhibit a consistent “EE1 → EE2 → Main” increase in Mean performance, in line with the elastic design described earlier.

On the efficiency side, DyPRSI-Main runs at 407.52 ms, nearly identical to BAAM (409.01 ms). Moreover, DyPRSI-EE1 and DyPRSI-EE2 reduce inference time to 235.48 ms and 243.80 ms, corresponding to approximately 42.4% and 40.4% lower latency than BAAM, respectively. The similar latency between EE1 and EE2 is expected because, in BAAM-style two-stage pipelines, the overall runtime is dominated by the RoI heads. For BAAM, the total 409.01 ms consists of 79.93 ms for backbone+BiFPN and 329.07 ms for the RoI heads. DyPRSI therefore gains most of its speed-up from lightweight branch predictors, whereas early-exiting on the trunk only saves a small amount of BiFPN computation (on the order of 10 ms), making the end-to-end latency of EE1 and EE2 close. While reducing the vertex count has little impact on forward inference time, it can substantially reduce post-processing overhead such as visualization.

**Figure 3:**
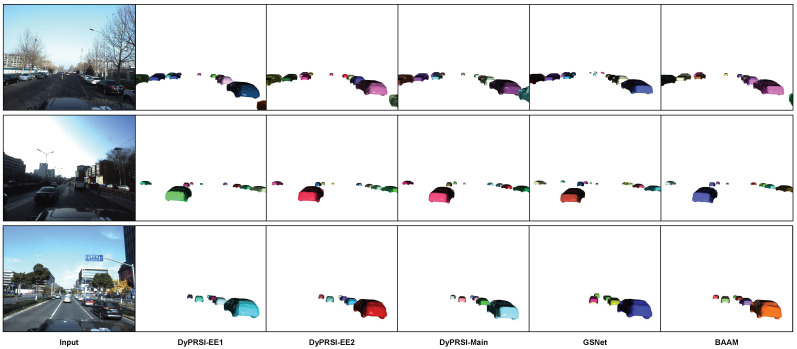
Qualitative comparison on ApolloCar3D. From left to right are the input image and reconstructed instance meshes produced by DyPRSI-EE1, DyPRSI-EE2, DyPRSI-Main, GSNet, and BAAM.

Figure 3 presents qualitative visual comparisons of DyPRSI-EE1, DyPRSI-EE2, DyPRSI-Main, GSNet, and BAAM on the ApolloCar3D dataset. Across the three representative scenes, the DyPRSI branches preserve consistent global layout and exhibit no obvious pose drift caused by early exiting. As the branch depth and mesh specification increase from EE1 to Main, local vehicle geometry becomes progressively more stable and detailed. In these examples, DyPRSI-Main yields poses and shapes closer to BAAM, whereas GSNet is more prone to visible shape or pose deviations. This suggests that the proposed branch hierarchy preserves global pose consistency reasonably well, while deeper branches mainly improve local geometric stability.

Overall, DyPRSI preserves the high-accuracy upper bound of the Main branch while offering substantial latency reductions via EE1/EE2, providing multiple practical accuracy–latency operating points under different compute budgets and deployment requirements.

### 4.4. Ablation Study

To evaluate how branch-specific keypoint head design affects DyPRSI’s multi-exit behavior, we compare two implementations: heatmap regression (HR) and coordinate classification (CC). With the backbone network and training setup kept identical, we consider three configurations: Default (HR for Main, CC for EE1/EE2), All-HR (HR for all branches) and All-CC (CC for all branches). In addition, the mesh specification of each branch is fixed to 488/828/1352 vertices for EE1/EE2/Main, respectively. The results are summarized in Table 3.

Table 3 reports a latency breakdown into the shared trunk (backbone+BiFPN) and RoI heads, together with the total inference time. We observe that the backbone + BiFPN cost is mainly determined by the exit depth, staying around 63 ms/71 ms/80 ms for EE1/EE2/Main, and remains nearly unchanged across different keypoint head choices. This indicates that the keypoint head has negligible impact on the computation of the shared trunk. In contrast, the dominant difference comes from the RoI heads: using HR incurs about 328–332 ms, whereas CC reduces it to 171–174 ms, nearly halving this component and substantially decreasing the total latency. For the early-exit paths, EE1 takes 396.02 ms under All-HR but drops to 236.03 ms under Default; similarly, EE2 decreases from 399.02 ms (All-HR) to 243.80 ms (Default), corresponding to an overall reduction of about 39–40%. Therefore, for early-exit branches targeting low latency, CC better matches the efficiency requirement.

In terms of accuracy, the benefit of HR over CC is generally limited for early-exit branches: the A3DP-Abs/Rel differences for EE1 and EE2 fluctuate within a small range, suggesting that HR provides only marginal gains while incurring a much higher computational cost on lightweight early-exit paths. In contrast, HR is more critical for the accuracy upper bound of the Main branch: compared with All-CC, the Main branch in Default improves A3DP-Abs and A3DP-Rel by 0.65 and 1.44, indicating that HR is more favorable for stable and high-accuracy reconstruction. Notably, even when both settings use HR for the Main branch, Default still outperforms All-HR, implying that switching EE branches from HR to CC may mitigate inter-branch interference during joint training and thereby positively impact the Main branch.

Overall, the keypoint head exhibits a clear branch-dependent behavior in DyPRSI: CC significantly reduces the RoI-head latency, enabling large speedups for EE branches with almost no change to the trunk cost, while HR helps preserve the accuracy upper bound on the Main branch at the expense of higher total inference time. Based on these observations, we adopt Default (Main-HR + EE1/EE2-CC) as the default configuration to maximize early-exit efficiency while maintaining a strong high-accuracy main path, thus better supporting DyPRSI’s resource-aware multi-exit reconstruction objective.

## 5. Conclusions

We propose DyPRSI, a dynamic-parameterized monocular vehicle 3D reconstruction framework for resource-aware spatial intelligence. DyPRSI inserts two early exits into a shared Res2Net-BiFPN trunk and associates each exit with a predefined mesh specification, so that a single model can provide multiple predefined accuracy–latency operating points for coarse-to-fine reconstruction. To better match the efficiency requirements of shallow branches, DyPRSI adopts lightweight coordinate-classification keypoint decoding in EE1 and EE2, while retaining heatmap-regression keypoint estimation in the Main branch to preserve the accuracy ceiling.

Experiments on ApolloCar3D show that DyPRSI-Main achieves competitive reconstruction accuracy compared with strong mesh-based baselines, while EE1 and EE2 substantially reduce end-to-end inference latency and remain effective as lower-cost alternatives under different resource requirements. Ablation results further indicate that the main speedup of the early-exit branches comes from the lightweight branch-specific keypoint heads, whereas the latency reduction gained from shallower trunk computation is relatively marginal. Meanwhile, using heatmap regression in the Main branch remains beneficial for maintaining stable high-accuracy reconstruction.

Overall, DyPRSI provides a practical paradigm for structured monocular vehicle reconstruction under different deployment budgets within a unified framework. Although the current study focuses on multiple predefined operating points rather than adaptive online exit selection, it establishes an effective basis for future extensions toward budget-aware policy design, broader object categories, and tighter integration with downstream spatial-intelligence tasks. 

## Figures and Tables

**Table 1:** Experimental environment parameters.

Equipment	Computer Configuration Parameters
Operating system	Linux
RAM	32 G
Type of operating system	Ubuntu20.04
CPU	Intel Core i7-12700K
GPU	RTX 4090 (24 GB) × 1
Development language	Python 3.9
Deep learning framework	PyTorch 1.10.1

**Table 2:** Main results on ApolloCar3D. We compare DyPRSI with GSNet and BAAM in terms of A3DP-Abs/A3DP-Rel accuracy and end-to-end inference time. DyPRSI provides three elastic branches (EE1/EE2/Main) with different compute–accuracy trade-offs.

Methods	A3DP-Abs Mean ↑	A3DP-Abs c-l ↑	A3DP-Abs c-s ↑	A3DP-Rel Mean ↑	A3DP-Rel c-l ↑	A3DP-Rel c-s ↑	Inf. Time (ms) ↓	Vertices
DyPRSI-EE1	21.31	42.89	19.27	17.08	38.89	13.06	235.48	488
DyPRSI-EE2	22.57	46.42	19.49	19.63	44.16	15.30	243.80	828
DyPRSI-Main	23.39	47.68	20.09	21.83	46.35	17.88	407.52	1352
GSNet	18.91	37.42	18.35	20.19	40.38	19.47	180.26	1352
BAAM	23.40	44.07	22.01	19.39	40.60	15.85	409.01	1352

↑ indicates that higher values are better. ↓ indicates that lower values are better.

**Table 3:** Ablation with inference-time breakdown on ApolloCar3D.

Model		A3DP-Abs-Mean ↑	A3DP-Rel-Mean ↑	Inf. Time (ms) ↓			Vertices
				Backbone + BiFPN	RoI Heads	Total	
DyPRSI (Default)	EE1-CC	21.31	17.08	63.39	172.64	236.03	488
	EE2-CC	22.57	19.63	71.75	172.05	243.80	828
	Main-HR	23.39	21.83	79.64	327.87	407.52	1352
DyPRSI (All-HR)	EE1-HR	21.56	17.20	63.62	332.40	396.02	488
	EE2-HR	22.66	19.73	71.29	327.73	399.02	828
	Main-HR	23.24	20.83	79.96	330.09	410.05	1352
DyPRSI (All-CC)	EE1-CC	21.54	17.29	63.21	172.03	235.24	488
	EE2-CC	22.29	19.10	71.68	171.34	243.03	828
	Main-CC	22.74	20.39	79.94	174.38	254.32	1352

↑ indicates that higher values are better. ↓ indicates that lower values are better.

## Data Availability

Restrictions apply to the availability of these data. The experiments in this study were conducted using the ApolloCar3D dataset (ApolloScape). Project information and toolkit resources are available at https://github.com/ApolloScapeAuto/dataset-api (accessed on 2 April 2026). Because direct public download of the full ApolloCar3D data was not consistently available at the time of access, the dataset is not redistributed by the authors. Access may require contacting the ApolloScape/ApolloCar3D maintainers or original dataset authors.
